# Anticoagulation Control with Acenocoumarol or Warfarin in Non-Valvular Atrial Fibrillation in Primary Care (Fantas-TIC Study)

**DOI:** 10.3390/ijerph18115700

**Published:** 2021-05-26

**Authors:** M. Rosa Dalmau Llorca, Carina Aguilar Martín, Noèlia Carrasco-Querol, Zojaina Hernández Rojas, Emma Forcadell Drago, Dolores Rodríguez Cumplido, Elisabet Castro Blanco, Alessandra Queiroga Gonçalves, José Fernández-Sáez

**Affiliations:** 1 Equip d’Atenció Primària Terres de l’Ebre, Institut Català de la Salut, 43500 Tortosa, Tarragona, Spain; rdalmau.ebre.ics@gencat.cat (M.R.D.L.); eforcadellg.ebre.ics@gencat.cat (E.F.D.); 2 Campus Terres de l’Ebre, Universitat Rovira i Virgili, 43500 Tortosa, Tarragona, Spain; elicasblan@gmail.com (E.C.B.); jfernandez@idiapjgol.info (J.F.-S.); 3 Primary Care Intervention Evaluation Research Group (GAVINA Research Group), 43500 Tortosa, Tarragona, Spain; caguilar.ebre.ics@gencat.cat (C.A.M.); drficf@gmail.com (D.R.C.); aqueiroga.ebre.ics@gencat.cat (A.Q.G.); 4 Unitat de Suport a la Recerca Terres de l’Ebre, Fundació Institut Universitari per a la Recerca a l’Atenció Primària de Salut Jordi Gol i Gurina (IDIAPJGol), 43500 Tortosa, Tarragona, Spain; 5 Unitat d’Avaluació, Direcció d’Atenció Primària Terres de l’Ebre, Institut Català de la Salut, 43500 Tortosa, Tarragona, Spain; 6 Hospital Universitari de Bellvitge, Institut Català de la Salut, 08907 Barcelona, Spain; 7 Unitat Docent de Medicina de Familia i Comunitària, Tortosa-Terres de l’Ebre, Institut Català de la Salut, 43500 Tortosa, Tarragona, Spain; 8 Unitat de Recerca, Gerència Territorial Terres de l’Ebre (Institut Catalá de la Salut), 43500 Tortosa, Tarragona, Spain

**Keywords:** atrial fibrillation, vitamin K antagonists, acenocoumarol, warfarin, time in therapeutic range

## Abstract

**Introduction:** The use of vitamin K antagonists (VKAs) in non-valvular atrial fibrillation (NVAF) is complicated due to the narrow therapeutic margin they present and their unpredictable dose–response relationship. Most studies are based on warfarin, with the results being extrapolated to acenocoumarol. However, studies comparing the two treatments in terms of the degree of anticoagulation control are scarce, justifying the present study. Main factors associated with poor control of time in therapeutic range (TTR) of anticoagulated patients are also studied. **Methods:** Cross-sectional study, with real-world data from patients treated in primary care (PC). Data were obtained from the System for the Improvement of Research in PC (SIDIAP) database, covering 60,978 NVAF-anticoagulated patients from 287 PC centres in 2018. Descriptive statistics were derived, and odds ratios were estimated by multivariate logistic regression. **Results:** 41,430 patients were considered: 93% were being treated with acenocoumarol and 7% with warfarin. There was no difference in poor control of TTR between the two types of VKA treatment, acenocoumarol and warfarin (38.9 vs. 38.4; *p* = 0.610). Poor anticoagulation control was mainly associated with advanced alcoholism (OR = 1.38), liver failure (OR = 1.37) and intracranial haemorrhage (OR = 1.35) as well as female sex, age < 60 years, cardiovascular history, diabetes mellitus and other variables. **Conclusions:** There is no association between poor anticoagulation control and the type of VKA treatment administered. Factors associated with poor control of TTR must be considered in clinical practice to improve control and decision-making.

## 1. Introduction

Thromboembolic complications of non-valvular atrial fibrillation (NVAF) are prevented by two large groups of oral anticoagulation drugs: vitamin K antagonists (VKAs) and direct-acting oral anticoagulants (DOACs). These treatments play a fundamental role, since they have been associated with a reduction in the risk of stroke, systemic embolism and mortality in patients with NVAF [1].

VKAs, such as acenocoumarol and warfarin, act by inhibiting gamma carboxylation, thus preventing the action of vitamin K-dependent factors II, VII, IX and X. They are characterized by having a tight therapeutic margin with a highly variable individual response, as well as dietary and drug interactions, which make periodic analytical controls necessary to monitor the level of anticoagulation [1]. They were the first anticoagulants to be used to treat NVAF, as well as the only therapeutic option until DOACs came onto the market a few years ago [2,3,4]. Even so, VKAs are still used throughout the world [2,5,6], and they are the treatment of first choice in Spain, according to the Ministry of Health [7]. The use of VKAs is complicated by a narrow therapeutic window and an unpredictable dose–response relationship, which lead to frequent bleeding complications or insufficient anticoagulation. Such variations in the response to the dose are markedly influenced by pharmacokinetic aspects, which are determined by genetic and environmental factors, and possibly others that have not yet been identified [8].

Both molecules act as vitamin K antagonists and have similarities and differences at various levels, particularly those of a pharmacokinetic nature acting at the level of genetic polymorphisms. Acenocoumarol and warfarin are not completely equivalent. One of the most relevant differences between them is their half-lives. Acenocoumarol has a 1.8 h half-life [9], while warfarin has a half-life of 24–33 h [10]. Furthermore, genetic polymorphisms affect drug metabolism differently depending on the type of VKA. The isoenzyme CYP2C9 may be more important for metabolising warfarin than acenocoumarol [11]. VKA use also differs from country to country: for example, the most widely used VKA in Spain and the Netherlands is acenocoumarol [12,13,14], whereas in the United States and other European countries it is warfarin [2,6]. In Spain, acenocoumarol is more widely used than warfarin probably because its use was authorized sooner, in 1956 [15], and because its shorter half-life facilitates faster control of its bleeding adverse effects.

Most of the scientific evidence related to this group of drugs comes primarily from studies of warfarin, the results of which are frequently generalized to acenocoumarol. However, few studies have been published that justify this generalization by comparing different aspects of the two drugs.

It is of fundamental importance to know which of the drugs provides the best control of anticoagulation over a particular period.

The degree of anticoagulation control of a patient is measured as their time in therapeutic range (TTR). One of the methods used for this purpose is the Rosendaal method, which enables the percentage of time the patient is within the therapeutic range to be calculated, with a TTR > 65% after six months of anticoagulation treatment being considered a good level of control [1]. Studies in our environment using this method show poor control, with a value of 39%, and no significant differences between the two types of VKA [16]. Poor control of anticoagulation is associated with an increase in the risk of stroke, bleeding and all-cause mortality [17,18]. The management of VKAs is associated with poor control routine, especially at the start of anticoagulation. Not all countries have the same degree of anticoagulation control. There is better control of anticoagulation with VKAs in Europe than in North America [19]. Given the evidence of poor control with VKAs in many countries and the greater use of acenocoumarol in Spain, although most studies are concerned with warfarin, it is essential to study the differences in the degree of control with both VKAs in our environment. Furthermore, in clinical practice, it is common to switch patients who do not achieve good control with acenocoumarol to warfarin treatment, with the aim of improving the anticoagulation control. The longer half-life and duration of action of warfarin can be erroneously associated with increased drug stability and potential better TTR control.

The objective of this study is to determine whether there are differences between acenocoumarol and warfarin in terms of their degrees of anticoagulation control. This was addressed by assessing the TTR and identifying the factors associated with poor VKA anticoagulation control in patients with NVAF.

## 2. Materials and Methods

### 2.1. Design and Study Population

**Figure 1:**
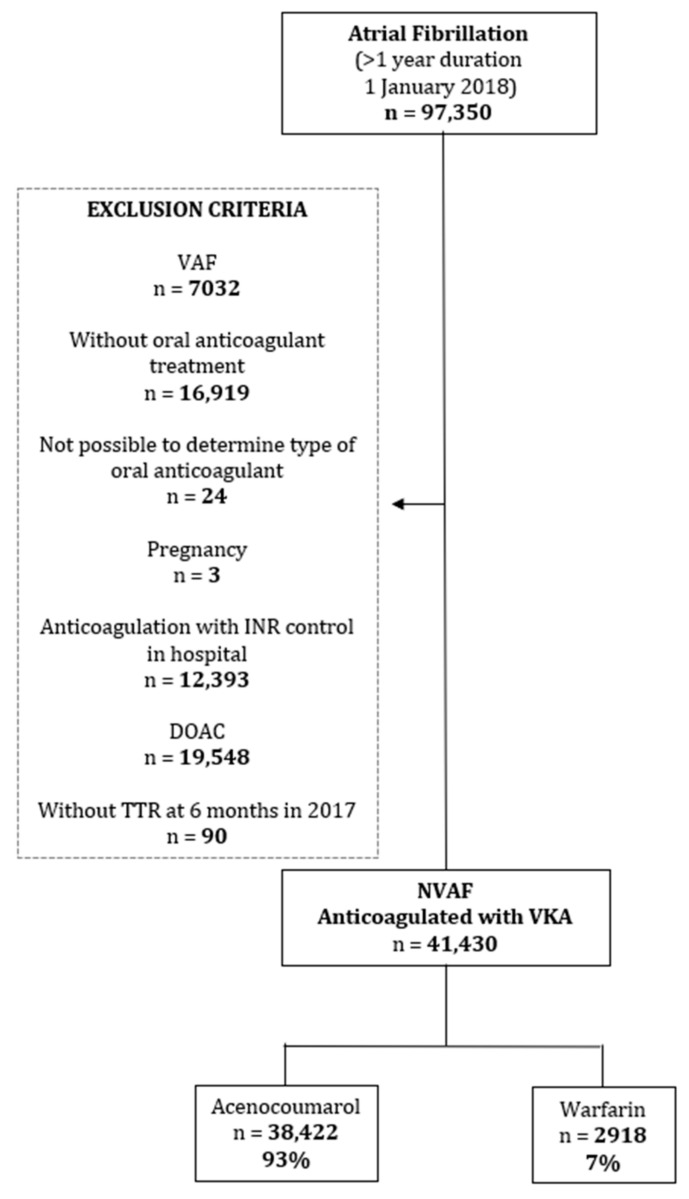
Flow diagram.

The present aim of the Fantas-TIC Study is to determine the degree of the antico-agulation control with acenocoumarol or warfarin in NVAF in Primary Care by as-sessing and comparing the TTR values. Factors associated with poor VKA anticoagula-tion control were also identified. To achieve the objective, a cross-sectional study was carried out with data obtained from the SIDIAP population database (Information System for Research in Primary Care) of 5,564,292 patients treated in primary care by the Catalan Institute of Health (ICS), representing 80% of the Catalan population. [20,21]. From the SIDIAP database, 97,350 patients with a diagnosis of AF for at least 12 months were identified (Figure 1). Patients included in the study had, on 1 January 2018, an active prescription for an anticoagulant. VKA anticoagulant treatments with acenocoumarol and warfarin were taken into account. Drug data was obtained on the basis of anatomic therapeutic chemical (ATC) codes [22].

SIDIAP includes anonymized clinical information from different data sources [20,21] such as PC electronic health records (EHRs). Since 2006, it has contained patients’ sociodemographic information, health conditions following the International Classification of Diseases (ICD) 10 codes [23], clinical parameters and general practitioner prescriptions. Since 2005, the SIDIAP database has included both laboratory and prescription data, with information based on ATC classification system codes [22] of the pharmaceutical products dispensed by Catalan community pharmacies.

### 2.2. Inclusion and Exclusion Criteria

We included primary care patients of the ICS receiving anticoagulant treatment with acenocoumarol or warfarin, diagnosed with NVAF one year before the study (as of 1 January 2018) and monitored in PC with at least six controls of the international normalized ratio (INR) during the year before the study. This criterion minimizes the variability of the INR that may occur when the treatment begins or when it is temporarily withdrawn. The inclusion and exclusion criteria are shown in Figure 1.

We excluded patients who were diagnosed with valvular FA (those with mitral stenosis or with a mechanical valve prosthesis) without anticoagulant treatment, those whose oral anticoagulant type at the time of the study could not be determined, pregnant women, patients with VKA controlled by the hospital INR, patients treated with DOACs and patients whose 6-month TTR the year prior to the study could not be calculated (Figure 1).

### 2.3. Study Variables

Main variable: Rosendaal time in therapeutic range (TTR) at 6 months in 2017 in patients anticoagulated with either of the VKAs acenocoumarol or warfarin, which are drugs included in the ATC code list [22]. Values of TTR < 65% at 6 months were considered to indicate poor anticoagulation control.

Secondary variables: sociodemographic variables, type of treatment, place of prescription, antecedent, cardiovascular antecedents, intracranial haemorrhage (ICH), morbidity, gastrointestinal bleeding (GIB), history of high risk of bleeding and other haemorrhages and scores calculated from real data of participants (CHA_2_DS_2_VASc and HAS_BLESD). Diseases were classified as specified in the ICD10 code list [23].

### 2.4. Statistical Analysis

Data were cleaned by checking the minimum and maximum values of the variables, as well as by an analysis of missing data. The Kolmogorov–Smirnov test of normality was performed on the 2017 6-month TTR variable to check whether it was normally distributed in both acenocoumarol and warfarin patients. 

Values of the 2017 6-month TTR variable were categorized into poor (TTR < 65%) and good (TTR ≥ 65%) anticoagulation control. Having cleaned the database, a descriptive analysis of poor control of TTR was carried out with respect to the type of VKA and the characteristics of the patients. Statistically significant differences between the categories of the variables studied according to the type of medication were tested using a two-proportion Z-test. To measure the association between the type of VKA used for TTR control and the other variables, and to study the factors related to poor control of anticoagulation with VKA, adjusted odd ratios (ORs) were estimated by multivariate logistic regression. For each type of VKA and for each value of HAS-BLED and CHA_2_DS_2_VASc, the mean TTR values and 95% confidence intervals (95% CIs) were calculated and plotted. 

Statistical analyses were carried out with Microsoft Excel 2010 and IBM SPSS Statistics version 20.0.

## 3. Results

The final population studied included a total of 41,430 patients, with NVAF criteria, anticoagulated with VKA and controlled under PC, of whom 38,422 (93%) were treated with acenocoumarol and 2918 (7%) with warfarin. Table 1 shows the characteristics of the patients studied.

There was no difference in the poor control of TTR between the two types of VKA treatment (acenocoumarol, 38.9 vs. warfarin, 38.4; *p* = 0.610) (Table 2). No statistically significant differences were observed in the bivariate analyses that compared poor control between the two types of VKA treatment for each patient variable studied (Table 2).

The results of the logistic regression did not show an association between the type of treatment and poor anticoagulation control when adjusted for the effects of the other variables (Table 3). Female sex and being younger than 60 years of age were patient variables associated to poor control. Other patient variables related to poor anticoagulation control were a cardiovascular history of peripheral arterial disease and ischaemic heart disease, as well as diabetes mellitus (DM), heart failure (HF) and renal insufficiency (RI), a history of ICH or GIB, a history of alcoholism and liver failure (LF). In contrast, oral anticoagulant prescription in PC and arterial hypertension were lightly protective factors of poor control. Receiving care outside of a PC centre was also related to poor anticoagulation control, i.e., patients whose care needs were provided at home or in institutional settings were more likely to show poor control than those who were attended in a PC centre.

**Figure 2:**
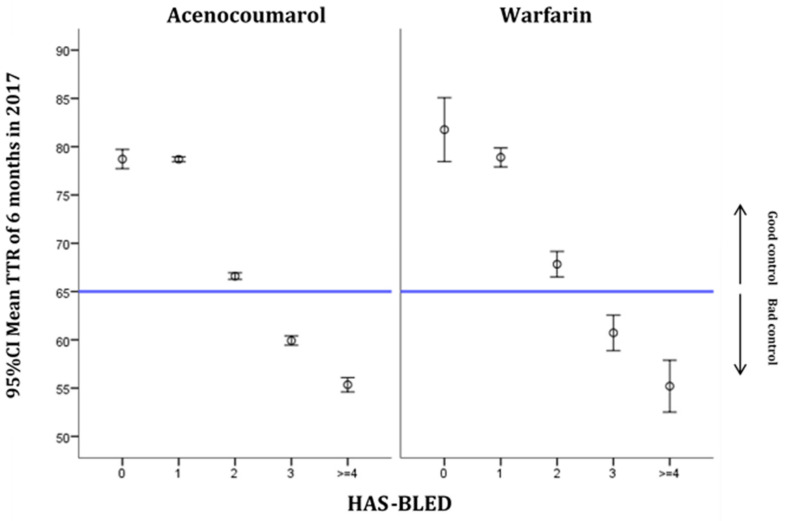
Distribution of TTR at 6 months in 2017 by HAS-BLED score and type of medication (mean and 95% CI).

The six-month TTR distributions based on HAS-BLED values by drug type are shown in Figure 2. The risk of bleeding became greater as the score increased. For both treatments, a score of 3 or more on this scale represents poor TTR control.

**Figure 3:**
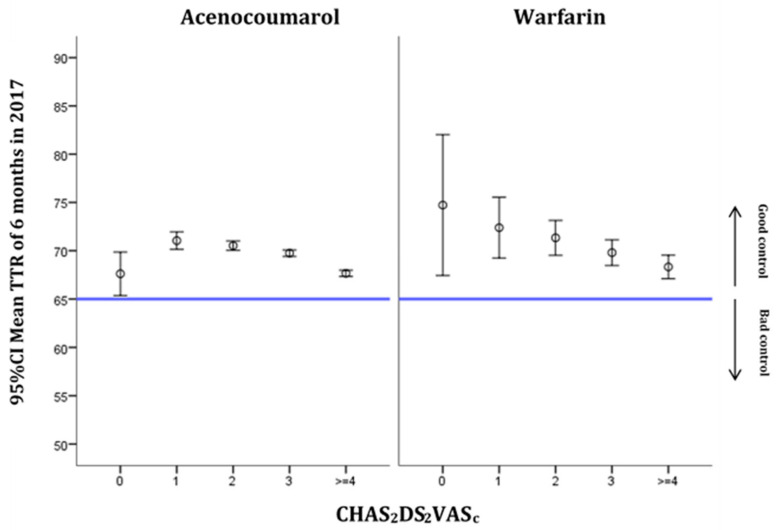
Distribution of TTR at 6 months in 2017 by CHA_2_DS_2_VASc score and type of medication (mean and 95% CI).

The six-month TRT distribution according to the CHA_2_DS_2_VASc scores is shown in Figure 3. The thromboembolic risk became greater as the scale score increased. The scores on the scale remained in the range of good TTR control for both VKAs. For acenocoumarol, all the CHA_2_DS_2_VASc scores were in the range of good TTR control, with anticoagulation control levels being worse when scores were 0 or ≥4. For warfarin, on the other hand, higher CHA_2_DS_2_VASc scores were linked to worse anticoagulation control.

## 4. Discussion

The present study was carried out with real-word data from anticoagulated NVAF patients who were followed up in PC and treated with a VKA, with the use of acenocoumarol clearly predominating over the use of warfarin. To our knowledge, it is the first study to show, with recent real-word data, that there is no association between poor anticoagulation control and the type of VKA treatment. Similarly, we found no difference in poor control by type of VKA treatment in relation to any other patient variables studied.

Examining the association of poor TTR control by type of VKA treatment when taking all other variables into account revealed no difference between the two treatments. However, an association was detected between poor control of anticoagulation with both acenocoumarol and warfarin and some patient characteristics. Female sex was associated with worse TRT control, as noted in a previous study [24]. This could be related to several factors, such as the more frequent occurrence in the female population of comorbidities such as dementia [24] and polypharmacy [25]. Specifically, polypharmacy in older people in Spain is more frequent in females [25] and is linked to an increased risk of drug interactions with anticoagulant treatment. At least 28 drugs are known to have interactions with acenocoumarol. These interactions are mainly pharmacokinetic, wherein acenocoumarol inhibits the cytochromes responsible for biotransformation [26]. Such drug interactions make it difficult to optimize doses and achieve good TRT control. The intensity of anticoagulation must be adapted to the established regime of medication at the start of anticoagulation. However, acute medications, such as Non-steroidal anti-inflammatory drugs (NSAIDs) and antibiotics that strongly disrupt coagulation for not inconsiderable periods of time, are less frequently taken into account.

Pharmacodynamic interactions are identical for acenocoumarol and warfarin [27], although the pharmacokinetic interactions do differ between these two drugs, since acenocoumarol is not metabolized by CYP3A4 as warfarin is [26]. Furthermore, the isoenzyme CYP2C9, the main cytochrome involved in the metabolism of coumarin anticoagulants, may be more important for the clearance of warfarin than of acenocoumarol. Some genetic polymorphisms related to this cytochrome increase the response to warfarin, but also to acenocoumarol [28]. According to recent studies, the correct dosage of acenocoumarol is largely determined by genetic variants, the age of the patient, sex, body mass index and the INR that it is wished to achieve [27]. These factors could explain 48% of the variation in dosage, and they need to be taken into account in order to improve anticoagulation control.

Regarding TTR control and patient age, our findings coincide with those of previous studies at the Spanish level, although the age ranges differ slightly; there is better INR control with both drugs in patients older than 60 years [24,29]. However, some published studies of the Chinese population indicate that, in patients treated with warfarin, being over 70 years of age is associated with poor control [30]. Such variation is probably related to the different geographic and cultural contexts and different sociodemographic characteristics, such as health care systems quality, organization, health care accessibility and domiciliary assistance [31]

Factors related to the risk of poor anticoagulation control included a cardiovascular history of peripheral artery disease and ischaemic heart disease, as well as bleeding (ICH or GIB). The morbidities that were related to poor control were DM, HF and RI, as well as a history of alcoholism and LF. Studies in our environment yielded similar results [32]. One of the patient variables with close association with poor TTR control was ICH. These patients should receive more attention and closer monitoring and would be candidates for switching to DOACs, on account of the ICH itself and because of their poor control of TTR. However, in some cases, patients with ICH could have advanced RI and be unable to make the switch to DOAC. 

Our study, similarly to previous ones, reveals an association between alcohol consumption and poor control of TTR. Alcohol interacts with treatment with VKAs, enhancing their effect, and is related to poor anticoagulation control, which increases the risk of bleeding [33]. In some countries, such as Sweden, where alcohol consumption is high, this is the main risk factor for poor individual TTR control (<60%) [34]. This study shows how in the group of patients with individual TTR values of <60%, the prevalence of alcohol consumption was three times that of the group with individual TTR values of >70% [34]. In the same way, the increase in the HAS-BLED score worsened the degree of control of TTR for scores of >3 for both VKAs, acenocoumarol and warfarin. This could be because the scale contains variables related to RI, LF and alcoholism. The increase in the score on the CHA_2_DS_2_VASc scale also yielded worse TTR values, but not to the extent as to be classified as poor control. This scale also contains variables, such as HF or DM, that have been associated with poorer TTR control or greater variability of INR [30,35]. On the other hand, differences in the confidence interval range could be related to the lower number of subjects treated with warfarin, and thus the lower number of subjects for both HAS-BLED and CHA_2_DS_2_VASc scoring zero compared to subjects treated with acenocoumarol.

One of the limitations of the study is its cross-sectional design, because cannot establish causality. However, it allows generating hypotheses, which opens the door to continuing studies of the similarities and differences between the two VKAs, given that most of the evidence is currently based on warfarin. This study considers data solely from PC and lacks hospital-based INR controls. This is another drawback, since it makes it difficult to generalize the results with confidence. On the other hand, the proportion of treatments with acenocoumarol (93%) is much higher than that of treatments with warfarin (7%) in our environment, although the use of population-based data means that the statistical power of the study is not compromised. The characteristics of the study population are similar to those of other studies of the Spanish population that claim to feature representative samples [24,29], and the population size considered enables the results from anticoagulated patients who have a diagnosis of NVAF and who are monitored in PC centres to be generalized. The degree of poor control of the participants treated with acenocoumarol and warfarin (38.9% and 38.4%, respectively) is similar to that of other studies in the Spanish setting, which have reported levels of 39.4%–47.3% of poor control with VKAs, with no differences between the treatments [29,32]. The few studies that have compared the differences in the degree of control in our setting reported Rosendaal TTR values of <65% for 32.1% and 31.7% of patients receiving acenocoumarol and warfarin, respectively. These values are lower than those of our study but, consistently with our findings, exhibit no differences between the treatment types [16,32].

The results provide evidence that the two VKAs are very similar with respect to their ability to provide anticoagulation control at six months and allow us to identify characteristics of patients that are related to the risk of poor control for both VKA treatments. Current observations suggest that patients not achieving good control with either of the two VKA drugs would not improve control by switching VKA drug. However, in these cases, patients could benefit from switching to DOAC treatment. 

These results will prompt further studies that will enable us to examine in even greater depth the differences between and similarities of these two VKAs, which continue to be the treatment of first choice for certain AF conditions. Studies of this nature could help to reduce thromboembolic and haemorrhagic risk while considering patient factors that are associated with poor control. Further studies must focus on identifying clinical support decision strategies to reduce poor TTR control degree and improving oral anticoagulant adequacy [36,37].

## Figures and Tables

**Table 1:** Characteristics of patients in the study.

Total	41,340
**Sex**	
Female	20,241 (49.0)
Male	21,099 (51.0)
**Age in Years. mean (SD)**	78.4 (9.0)
**Type of AVK**	
Acenocoumarol	38,422 (92.9)
Warfarin	2918 (7.1)
**Oral Anticoagulation Prescription in Primary Care**	33,185 (80.3)
**Cardiovascular History**	
Peripheral Arteriopathy	2698 (6.5)
Ischaemic Heart Disease	7494 (18.1)
Aortic Atheromatosis	399 (1.0)
Ischaemic Stroke or Transient Ischaemic Attack	6421 (15.5)
**Intracranial Haemorrhage**	363 (0.9)
**Morbidity**	
Diabetes Mellitus	13,552 (32.8)
Arterial Hypertension	33,284 (80.5)
Heart Failure	10,806 (26.1)
Renal Insufficiency	12,303 (29.8)
**Bleeding Risk History**	
Alcohol	1721 (4.2)
Intracranial Aneurysm	27 (0.1)
Portal Hypertension	80 (0.2)
Liver Failure	263 (0.6)
Hereditary Telangiectasia	2 (0.0)
Active Aortic Aneurysm and Dissection	612 (1.5)
Intestinal Angiodysplasia	92 (0.2)
Haemorrhages Other Than Gastrointestinal and Intracranial	567 (1.4)
**Gastrointestinal Bleeding**	3272 (7.9)
**Scores**	
CHA_2_DS_2_VASc	
0	404 (1.0)
1	2104 (5.1)
2	7246 (17.5)
3	14,656 (35.5)
≥4	16,930 (41.0)
HAS-BLED	
0	953 (2.3)
1	14,548 (35.2)
2	15,075 (36.5)
3	7754 (18.8)
≥4	3010 (7.3)
**eGFR mL/min/1.73 m^2^**	61.2 (18.9)
**Patients Treated Outside Primary Care Centre**	
Treated at Home	4892 (11.8)
Treated in an Institution	1636 (4.0)

Quantitative variables are expressed as mean (standard deviation) and qualitative variables as n (%).

**Table 2:** Description of poor control with respect to TTR < 65% at 6 months by type of treatment (acenocoumarol and warfarin) and patient characteristics.

	Acenocoumarol			Warfarin			
	Total	TTR < 65%	%	Total	TTR < 65%	%	^a^ *p*
**Total**	**38,422**	**14,944**	**38.9**	**2918**	**1121**	**38.4**	**0.610**
**Sex**		
Female	18,808	7626	40.5	1433	587	41.0	0.757
Male	19,614	7318	37.3	1485	534	36.0	0.299
**Age (years)**		
<60	862	370	42.9	72	27	37.5	0.371
60–69	4281	1610	37.6	397	145	36.5	0.670
70–79	12,558	4691	37.4	1085	390	35.9	0.357
≥80	20,721	8273	39.9	1364	559	41.0	0.440
**Oral Anticoagulant Prescription in Primary Care**		
Yes	30,938	11,964	38.7	2247	866	38.5	0.902
No	7484	2980	39.8	671	255	38.0	0.357
**Cardiovascular History**		
Peripheral Arteriopathy	2492	1088	43.7	206	89	43.2	0.899
No	35,930	13,856	38.6	2712	1032	38.1	0.598
Ischaemic Heart Disease	6951	2855	41.1	543	222	40.9	0.931
No	31,471	12,089	38.4	2375	899	37.9	0.588
Aortic Atheromatosis	354	139	39.3	45	16	35.6	0.631
No	38,068	14,805	38.9	2873	1105	38.5	0.649
Ischaemic Stroke or TIA	5810	2351	40.5	611	252	41.2	0.709
No	32,612	12,593	38.6	2307	869	37.7	0.367
**Intracranial Haemorrhage**	334	156	46.7	29	16	55.2	0.381
No	38,088	14,788	38.8	2889	1105	38.2	0.539
**Morbidity**		
Diabetes mellitus	12,549	5293	42.2	1003	406	40.5	0.294
No	25,873	9651	37.3	1915	715	37.3	0.975
Arterial Hypertension	30,947	12,047	38.9	2337	913	39.1	0.894
No	7475	2897	38.8	581	208	35.8	0.159
Heart Failure	9947	4320	43.4	859	363	42.3	0.506
No	28,475	10,624	37.3	2059	758	36.8	0.653
Renal Insufficiency	11,346	4752	41.9	957	417	43.6	0.309
No	27,076	10,192	37.6	1961	704	35.9	0.124
**Bleeding Risk History**		
Alcohol	1564	711	45.5	157	71	45.2	0.955
No	36,858	14,233	38.6	2761	1050	38.0	0.542
Intracranial Aneurysm	26	12	46.2	1	1	100.0	0.290
No	38,396	14,932	38.9	2917	1120	38.4	0.598
Portal Hypertension	72	27	37.5	8	4	50.0	0.491
No	38,350	14,917	38.9	2910	1117	38.4	0.585
Liver Failure	242	116	47.9	21	11	52.4	0.696
No	38,180	14,828	38.8	2897	1110	38.3	0.579
Hereditary Telangiectasia	1	0	0.0	1	1	100.0	0.157
No	38,421	14,944	38.9	2917	1120	38.4	0.593
Active Aortic Aneurysm and Dissection	558	207	37.1	54	27	50.0	0.062
No	37,864	14,737	38.9	2864	1094	38.2	0.444
Intestinal Angiodysplasia	85	40	47.1	7	4	57.1	0.608
No	38,337	14,904	38.9	2911	1117	38.4	0.590
Haemorrhages Other Than Gastrointestinal and Intracranial	523	194	37.1	44	16	36.4	0.923
No	37,899	14,750	38.9	2874	1105	38.4	0.617
**Gastrointestinal Bleeding**	3028	1252	41.3	244	101	41.4	0.989
No	35,394	13,692	38.7	2674	1020	38.1	0.581
**Scores**		
CHA_2_DS_2_VASc		
0	366	137	37.4	38	13	34.2	0.696
1	1939	683	35.2	165	54	32.7	0.519
2	6722	2402	35.7	524	170	32.4	0.129
3	13,698	5148	37.6	958	382	39.9	0.157
≥4	15,697	6574	41.9	1233	502	40.7	0.424
HAS-BLED		
0	872	168	19.3	81	11	13.6	0.210
1	13,530	2387	17.6	1018	190	18.7	0.410
2	14,065	6285	44.7	1010	443	43.9	0.611
3	7180	4210	58.6	574	320	55.7	0.177
≥4	2775	1894	68.3	235	157	66.8	0.648
eGFR mL/min/1.73 m^2^		
< 15	270	172	63.7	41	23	56.1	0.348
15–29	1678	825	49.2	164	84	51.2	0.616
30–49	7570	3292	43.5	639	274	42.9	0.766
≥ 50	25,887	9537	36.8	1926	679	35.3	0.164
Lost	3017	1118	37.1	148	61	41.2	0.307
**Patients Treated Outside Primary Care Centre**		
Treated at Home	4504	2149	47.7	388	184	47.4	0.912
No	33,918	12,795	37.7	2530	937	37.0	0.491
Treated in an Institution	1546	756	48.9	90	48	53.3	0.414
No	36,876	14,188	38.5	2828	1073	37.9	0.575

**^a^** Two-proportion Z-test.

**Table 3:** Association of poor control of TTR by treatment, adjusted for other patient variables.

	OR_adj_	95% CI	*p*
**Type of anticoagulant**			
Acenocoumarol	**1**		
Warfarin	0.96	0.88–1.03	0.268
**Sex**			
Female	1		
Male	0.87	0.83–0.91	**<0.001**
**Age (years)**			
<60	1		
60–69	0.82	0.71–0.95	**0.010**
70–79	0.78	0.68–0.90	**<0.001**
≥80	0.82	0.72–0.94	**0.004**
**Oral Anticoagulant Prescription in Primary Care**	0.95	0.90–1.00	**0.036**
**Cardiovascular History**			
Peripheral Arteriopathy	1.17	1.08–1.27	**<0.001**
Ischaemic Heart Disease	1.08	1.02–1.13	**0.006**
Aortic Atheromatosis	0.96	0.79–1.18	0.723
Ischaemic Stroke or Transient Ischaemic Attack	1.04	0.98–1.10	0.213
**Intracranial Haemorrhage**	1.35	1.10–1.67	**0.004**
**Morbidity**			
Diabetes Mellitus	1.19	1.14–1.24	**<0.001**
Arterial Hypertension	0.95	0.90–1.00	**0.034**
Heart Failure	1.18	1.12–1.23	**<0.001**
Renal Insufficiency	1.13	1.08–1.18	**<0.001**
**Bleeding Risk History**			
Alcohol	1.38	1.25–1.53	**<0.001**
Intracranial Aneurysm	1.36	0.63–2.91	0.436
Portal Hypertension	0.81	0.51–1.29	0.372
Liver Failure	1.37	1.07–1.77	**0.014**
Hereditary Telangiectasia	1.61	0.10–25.91	0.736
Active Aortic Aneurysm and Dissection	1.04	0.88–1.23	0.673
Intestinal Angiodysplasia	1.39	0.92–2.11	0.116
Haemorrhages Other Than Gastrointestinal and Intracranial	0.89	0.75–1.06	0.202
**Gastrointestinal Bleeding**	1.11	1.03–1.20	**0.005**
**Patients Treated Outside Primary Care Centre**			
Treated at Home	1.33	1.25–1.42	**<0.001**
Treated in an Institution	1.35	1.22–1.50	**<0.001**

ORadj: Adjusted ODDS Ratio for all model variables. Bold values: statistically significant values.

## Data Availability

The data that support the findings of this study were obtained from the SIDIAP database (Information System for Research in Primary Care). This database is representative of the Catalan population. Restrictions apply to the availability of these data, which were used under license for this study. The authors have no authorization to share the data.
